# The Classification of Medicines

**Published:** 1879-09

**Authors:** 


THE CLASSIFICATION OF MEDICINES.—Dr. Buchheim, of Glessen urges, in the *Archev de Pharmacie*, that the time has come when a new pharmarcognostic system of classification of drugs should be adopted. It is an error, he thinks, to suppose that the classification of drugs is a matter of small importance. Correct and wide views of an art or science depend very largely on the stand-point from which we regard the facts, and the manner in which they are marshalled before us. It is, therefore, of primary importance that the arrangement of our drugs should follow a scientific principle.

So long as physicians and pharmaceutists gathered their plants themselves, the method of classifying the materia medica according to a botanic system had its advantages. But the parts of plants used as drugs have no connection with their botanic origin, and, therefore, such an arrangement can have no pretension to be a scientific system. "Only the fact that no other classification appeared more perfect, can explain how such writers as Hanbury and Fluckger should have retained this system in their excellent Pharmacographia."

Other recent authors have endeavored to classify drugs as roots, barks, seeds, leaves, etc., and some have abandoned all system at all, except the alphabetic arrangement. But, according to Buckheim, the circumstances of the origin of a drug are of less practical importance than its condition when it comes before us. An official drug may be very truly the genuine plant which it professes to be, or the root or the seed, as the case may be, of that particular plant, but it may, nevertheless, be a very poor sample—in a sense, a very ungenuine drug. In brief, the time has come when we should look on cinchona bark merely as an impure quinine, on opium as an impure morphia, and so on. Therefore, Dr. Bucheim proposes to arrange medicines into groups, according to their active principles, and he argues that, by following such a principle of classification, we should be on the right road to ascertain with more exactness the true active principles of all drugs. He then gives a sketch of a classification on the principles he suggests.

I. Hydrocarbon group.—(*a*) Starches; (*b*) sugars; (c) gums and vegetable mucilages.

II. Albumens and their derivatives—(*a*) Albumens; (*b*) albuminoids; (*c*) putrid matters.

III. Glycerides—(*a*) Olive oil group (including besides the fatty oils, such fruits as almonds, poppies, cacao, etc., which depend for their properties on their fatty characteristics. Wax and spermaceti, though not glycerides, would appear as a supplement to this group). (*b*) Croton oil group (including castor oil and the other oils which have an action other than the merely nourishing properties of the oils of the preceding group).

IV. The Cardol group (Cardol, C. H. O.—The anacardium, Cayenne, ginger, etc.

V. The Mustard Oil group.

VI. The Cantharidin group.

VII. The Anhydrid Acids—(*a*) Euphobin and ambydrides; (*b*) convolvulin acid anhydrides.

VIII. The Aloctin group.

IX. Cathartic Acid group.

X. Filicic Acid group.

XI. The Tannic Acid group.

XII. The Alkaloid Group.—(*a*) The piperines; (*b*) the caffeines; (*d*) the coniines; (*e*) the strychnines; (*f*) the morphines (*g*) the atropines; (*h*) pilocarpine; (*i*) physostigmine; (*k*) nicotine ;(*l*) emetine; (*m*) the aconitines; (*n*) the veratrines; (*o*) colchicine.

XIII. The Glycoside Group.—(*a*) The digitaline ; (*b*) The digitalins; (*b*) the saponine.

XIV. The Bitter Principle group.

XV. The Ethereal Oil group.

XVI. Officinal Resins.

XVII. Officinal Coloring Matters.—*Chemist and Druggist.*

